# Supplementary material for: Systematic analysis and prediction of genes associated with monogenic disorders on human chromosome X
Source: Nat Commun. 2022 Nov 2;13:6570. doi: 10.1038/s41467-022-34264-y (PMC9630267; doi:10.1038/s41467-022-34264-y)
Supplement: Supplementary file 1 — Supplementary Information [file 41467_2022_34264_MOESM1_ESM.pdf]

Supplementary information

Systematic analysis and prediction of genes associated with monogenic disorders on human chromosome X

Elsa Leitão, Christopher Schröder, Ilaria Parenti, Carine Dalle, Agnès Rastetter, Theresa Kühnel, Alma Kuechler, Sabine Kaya, Bénédicte Gérard, Elise Schaefer, Caroline Nava, Nathalie Drouot, Camille Engel, Juliette Piard, Bénédicte Duban-Bedu, Laurent Villard, Alexander P.A. Stegmann, Els K. Vanhoutte, Job A.J Verdonschot, Frank J. Kaiser, Frédéric Tran Mau-Them, Marcello Scala, Pasquale Striano, Suzanna G.M. Frints, Emanuela Argilli, Elliott H. Sherr, Fikret Elder, Julien Buratti, Boris Keren, Cyril Mignot, Delphine Héron, Jean-Louis Mandel, Jozef Gecz, Vera M. Kalscheuer, Bernhard Horsthemke, Amélie Piton, Christel Depienne

Supplementary Figures

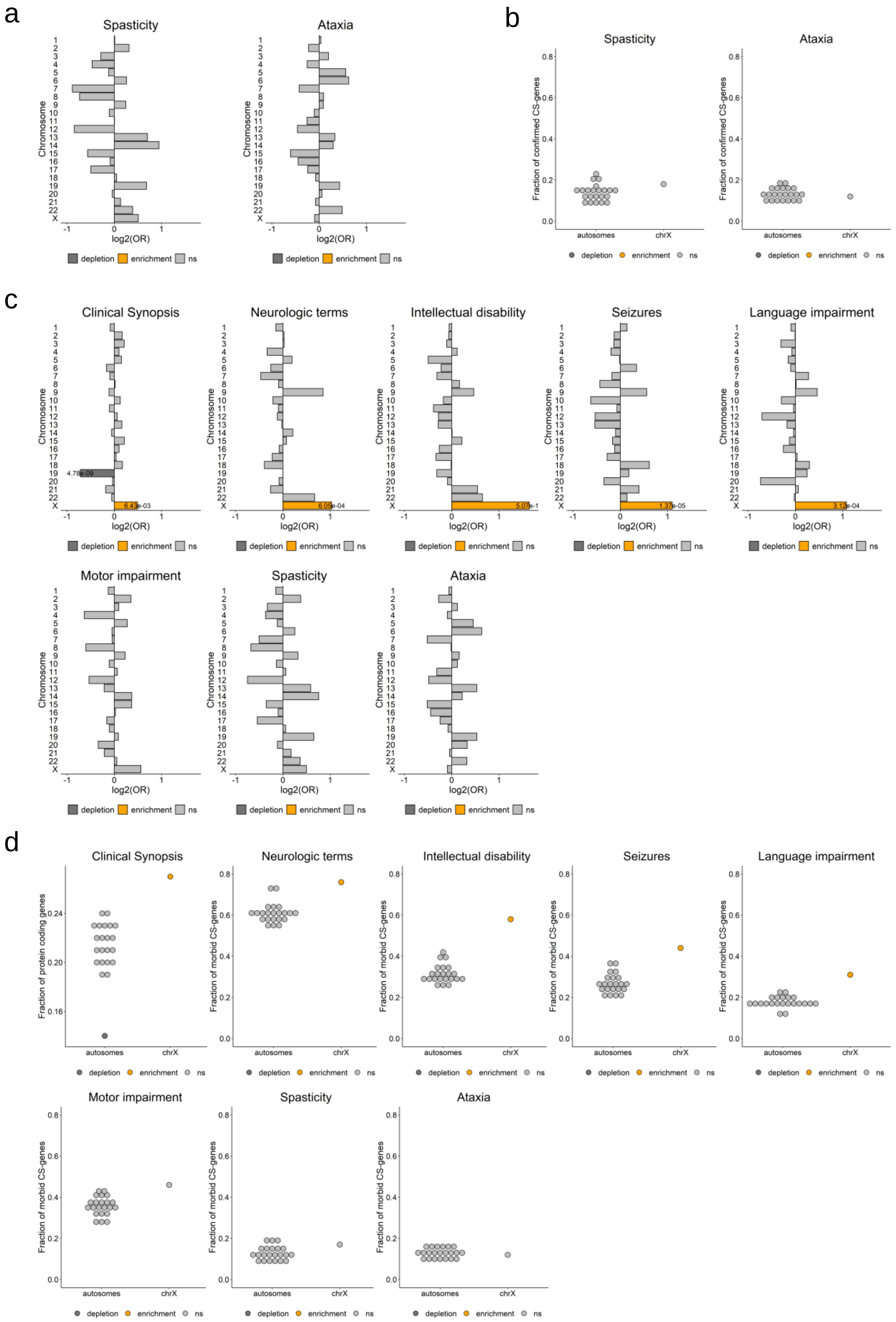

Supplementary Fig. 1 (legend next page)

Supplementary Fig. 1: Chromosome enrichment/depletion of genes associated with OMIM Clinical Synopsis features (CS-genes). a-b, Genes associated with motor impairment, spasticity or ataxia are not enriched nor depleted in specific chromosomes. **a,** Per chromosome enrichment/depletion of CS-genes having specific neurologic features described in the Clinical Synopsis data (Fisher's test (two-sided) followed by Bonferroni correction for multiple testing across both chromosomes and phenotypes). **b,** Fraction of CS-genes that contain specific neurologic terms in the associated Clinical Synopsis data. **c-d,** Comparisons of morbid genes with Clinical Synopsis information between chromosomes show similar results. **c,** Per chromosome enrichment/depletion of i) protein-coding genes in morbid genes with at least one associated phenotype comprising Clinical Synopsis data (morbid CS-genes) (first graph) and ii) morbid CS-genes in genes with non-specific or specific neurologic features described in the Clinical Synopsis data (other graphs). Only significant p-values are shown (Fisher's test (two-sided) followed by Bonferroni correction for multiple testing). **d,** Fraction of i) morbid CS-genes among protein-coding genes or ii) morbid CS-genes that contain non-specific or specific neurologic terms in the associated Clinical Synopsis data. **a-d,** Yellow, enrichment; dark-grey, depletion; light-grey, not significant (ns). Specific neurologic terms: intellectual disability, seizures, language impairment, motor impairment, spasticity and ataxia. Synonymous terms/sentences were used in OMIM searches (Supplementary Data 3). Source data are provided as a Source Data file.

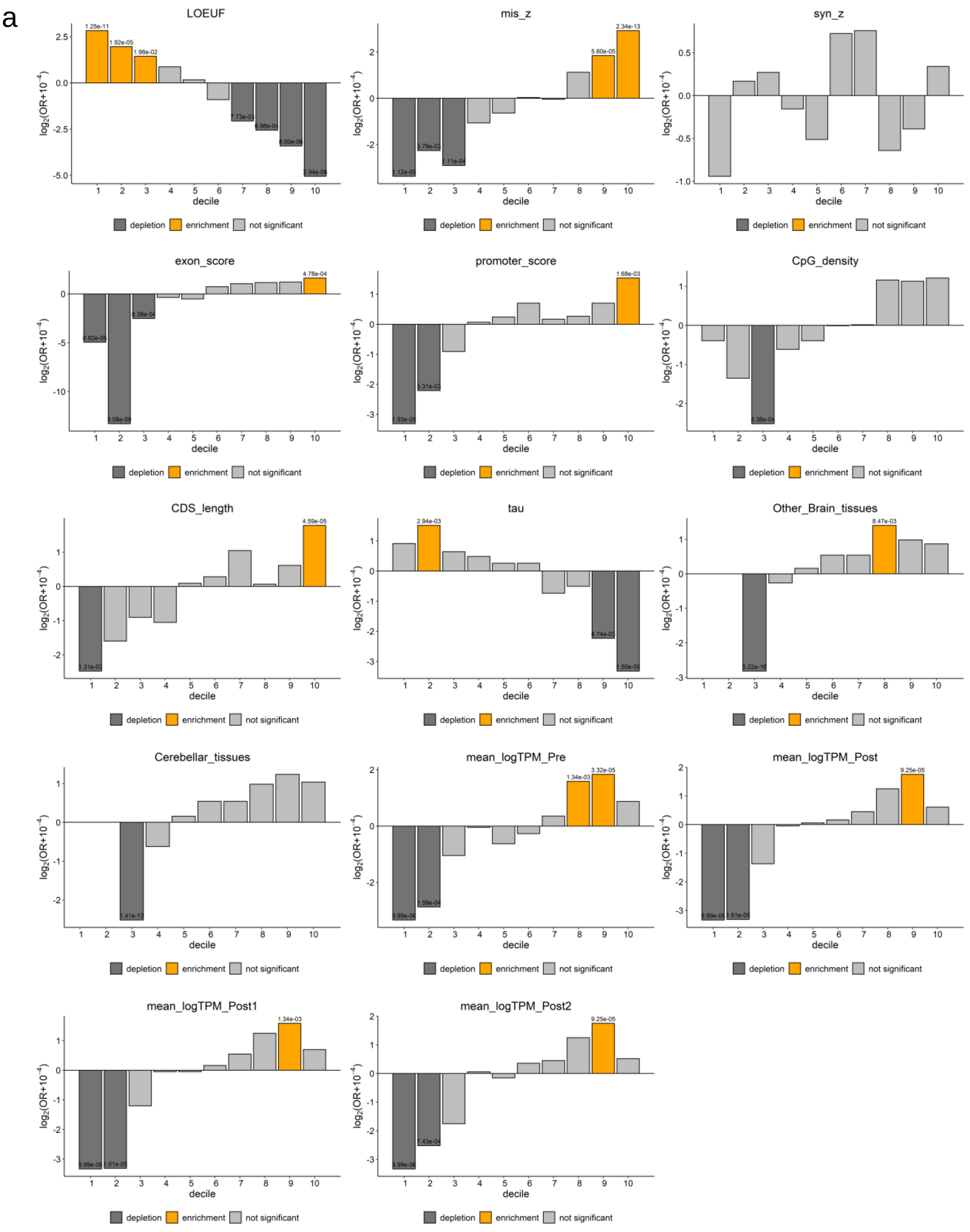

Supplementary Fig. 2 (continues next page)

b

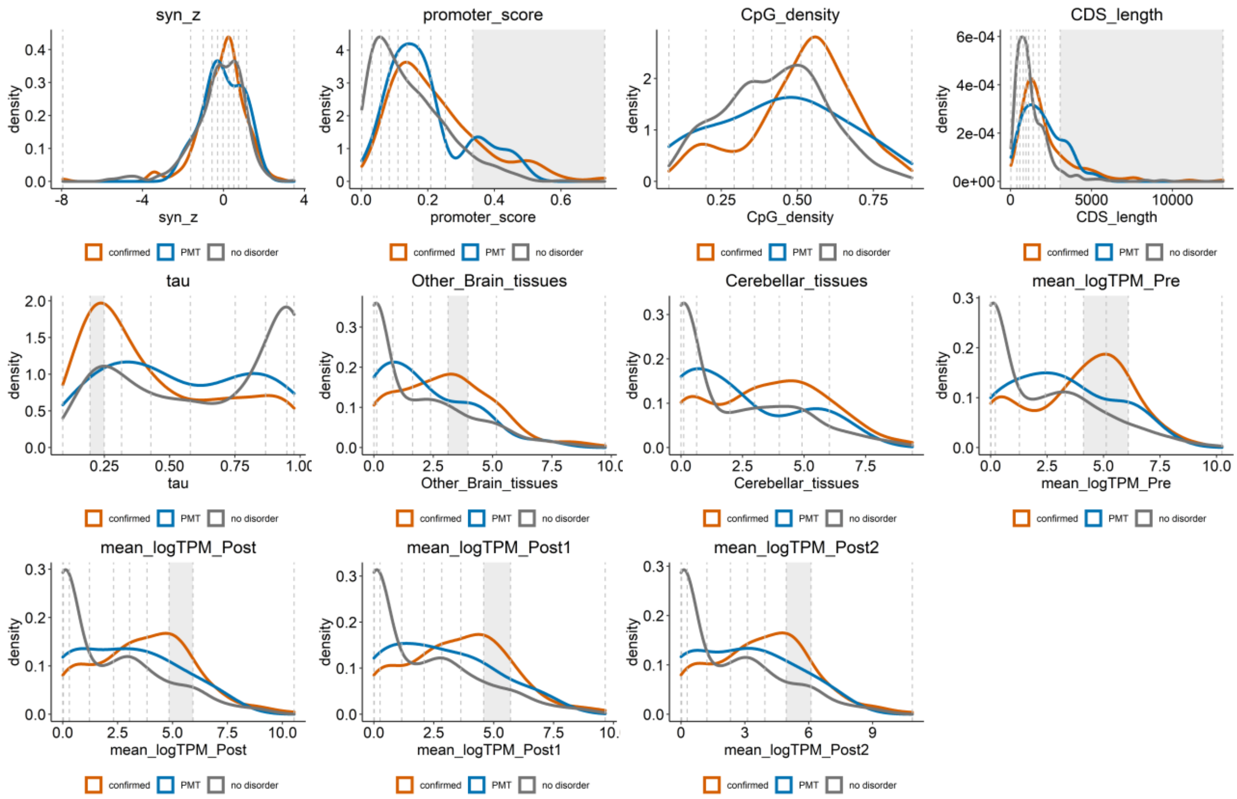

Supplementary Fig. 2: Analyses of continuous variable distributions. a, Barplots showing the enrichment of confirmed genes in each decile of continuous variable distributions. Yellow, enrichment; dark-grey, depletion; light-grey, not significant. Only significant p-values are shown (Fisher's test (two-sided) followed by Bonferroni correction for multiple testing, across both variables and deciles). **b,** Density plots showing the distribution of continuous variables according to gene group. Genes associated with at least one monogenic disorder (confirmed genes, orange), genes with provisional associations or associated with susceptibility factors to multifactorial disorders or with traits (PMTs, blue), and genes without associated phenotypes (no-disorder genes, grey). Vertical dashed lines separate deciles of the overall distribution. Grey areas depict deciles for which confirmed disease-causing genes are enriched (related to panel a). Source data are provided as a Source Data file.

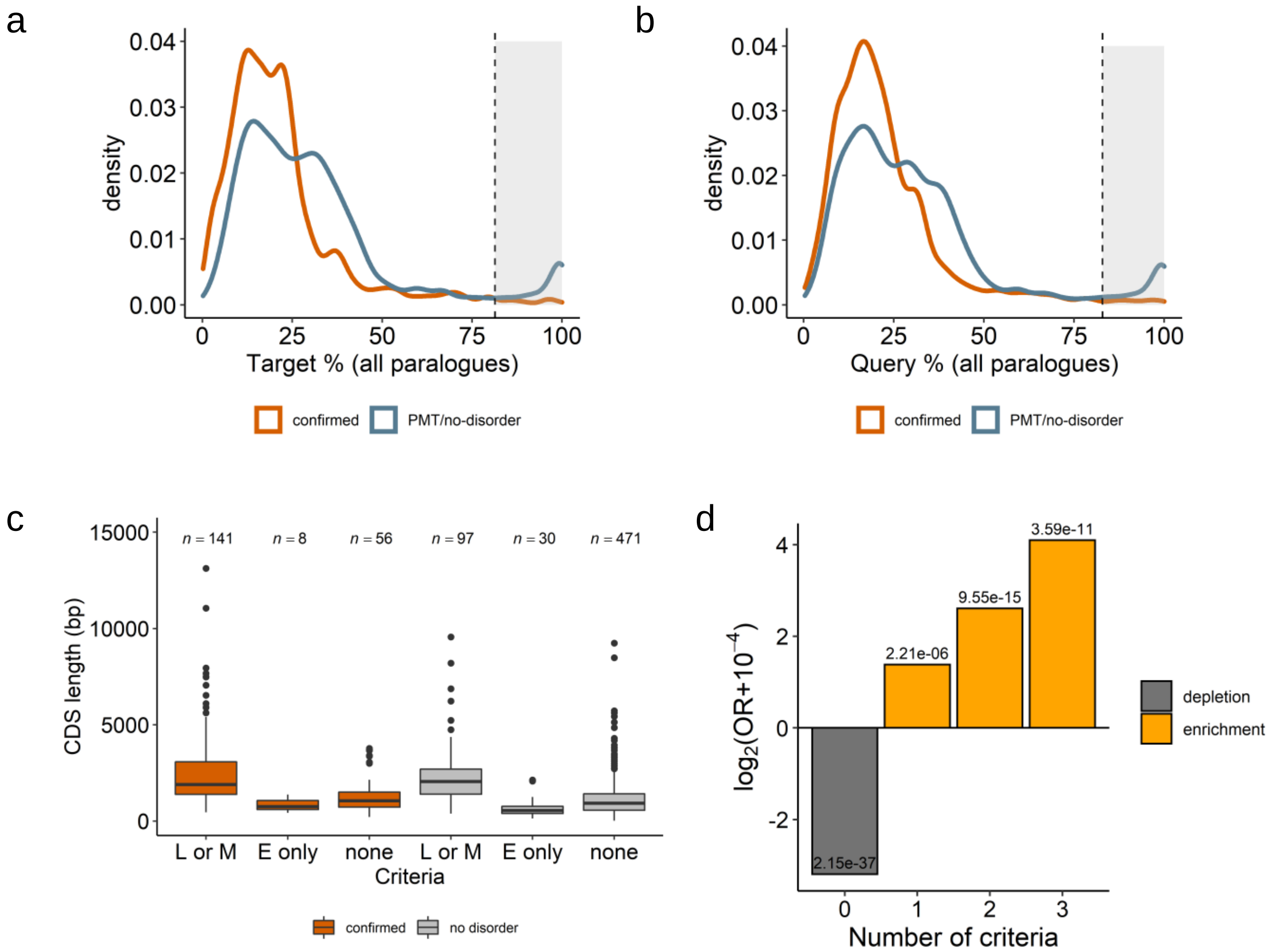

Supplementary Fig. 3: a and b, Paralogues of protein-coding genes on chrX. Density plots showing the distribution of target (**a**) and query (**c**) percentage of identity for all paralogues of protein-coding genes on chrX according to gene group. Vertical dashed lines depict the 95th percentile of the overall distribution (81.4 and 83.9% for target and query percentages of identity, respectively), and grey areas mark paralogues above the 95th percentiles. **c,** Boxplot showing a bias of LOEUF and misZ criteria against genes with smaller coding-sequence (CDS) length. Box plot elements are defined as follows: center line: median; box limits: upper and lower quartiles; whiskers: 1.5× interquartile range; points: outliers. **d,** Enrichment of confirmed genes in genes meeting at least one of the L, M or E criteria. Yellow, enrichment; dark-grey, depletion; light-grey, not significant. Significant p-values are shown (Fisher's test (two-sided) followed by Bonferroni correction for multiple testing). Source data are provided as a Source Data file.

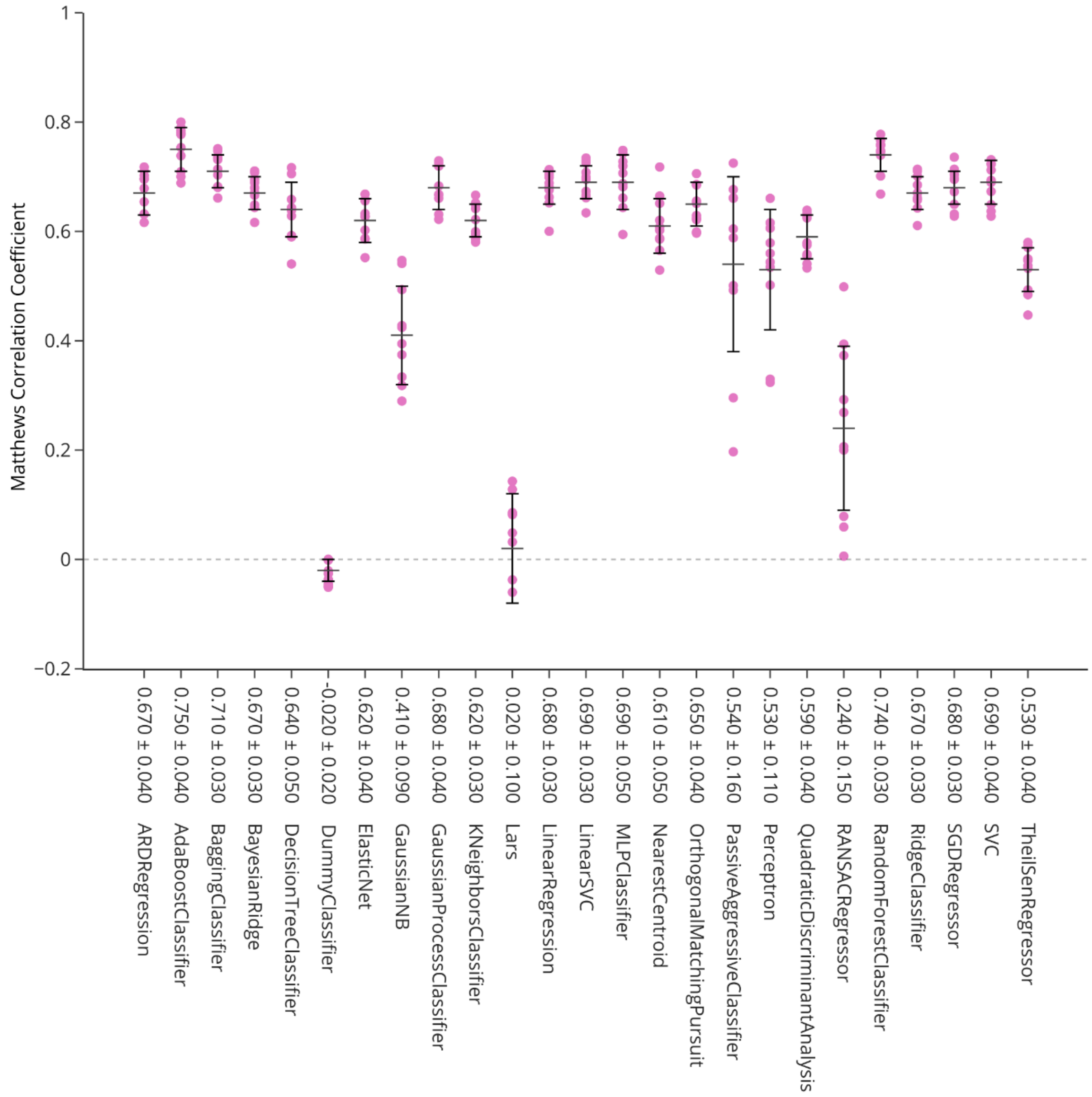

Supplementary Fig. 4: Mean (black middle line) Matthews Correlation Coefficient (MCC) of each 10-fold cross validation (purple points; *n* = 10) and their standard deviation (black whiskers) obtained for the 25 tested machine learning classifiers. Source data are provided as a Source Data file.

a

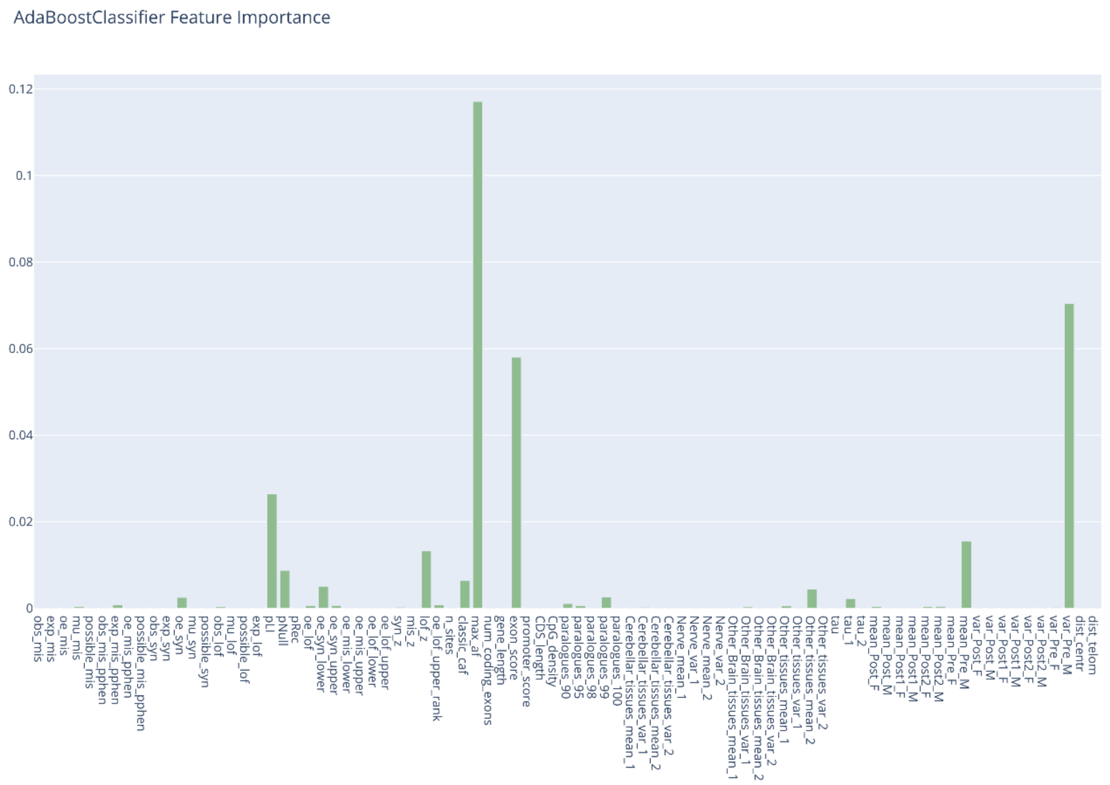

b

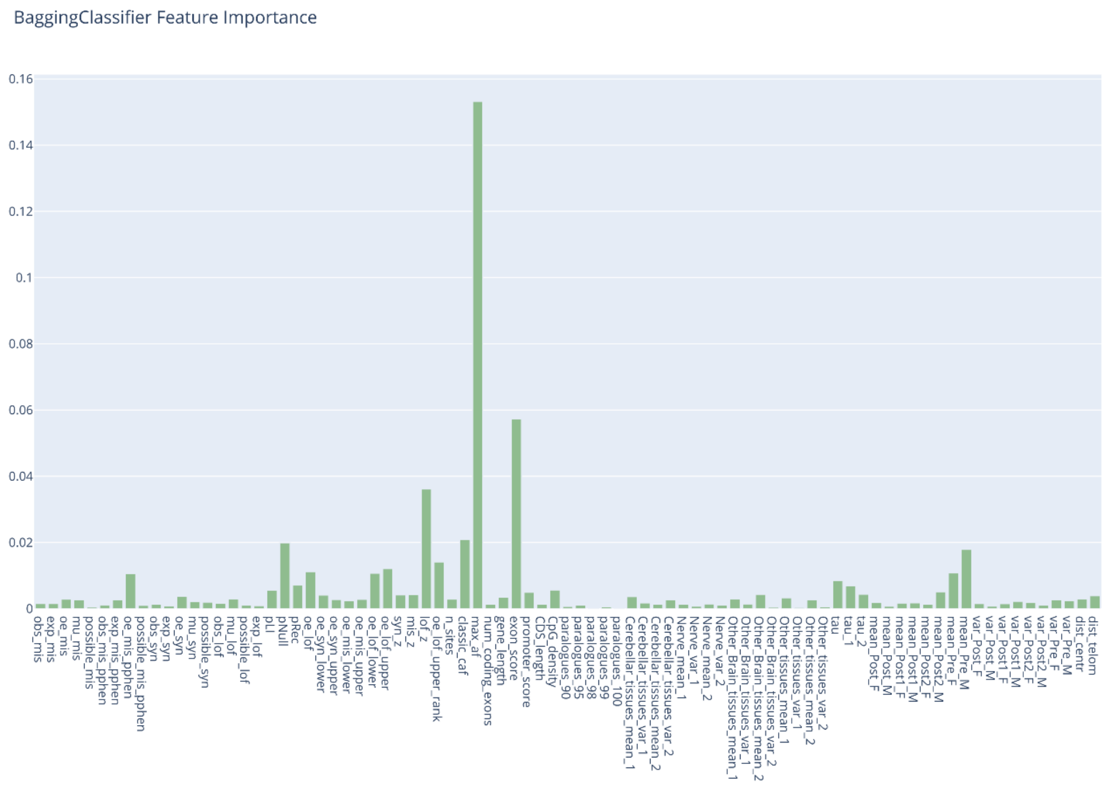

Supplementary Fig. 5 (continue next page)

c

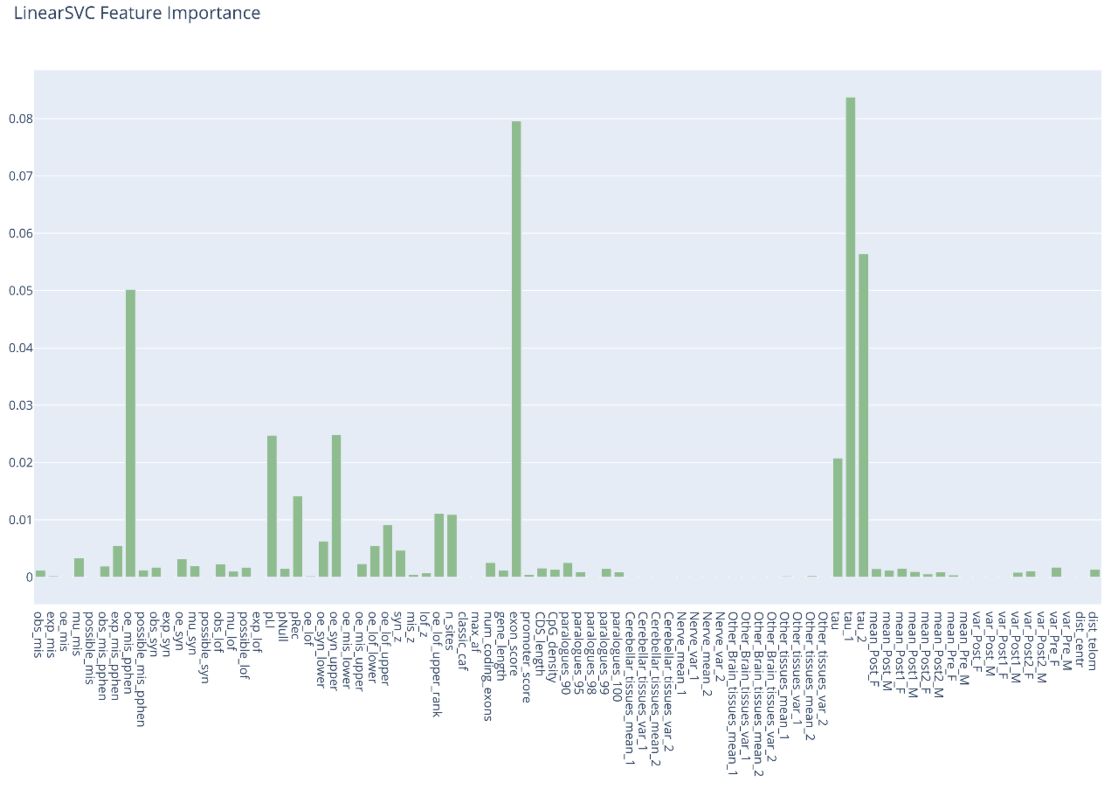

d

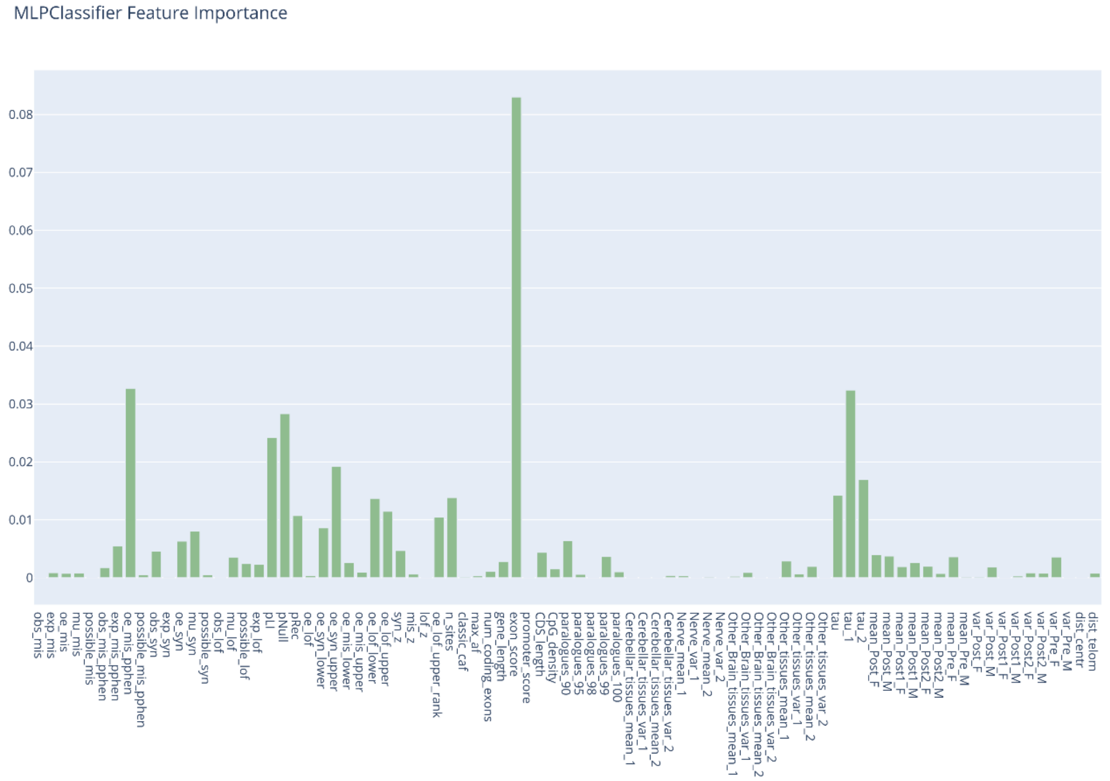

Supplementary Fig. 5 (continue next page)

e

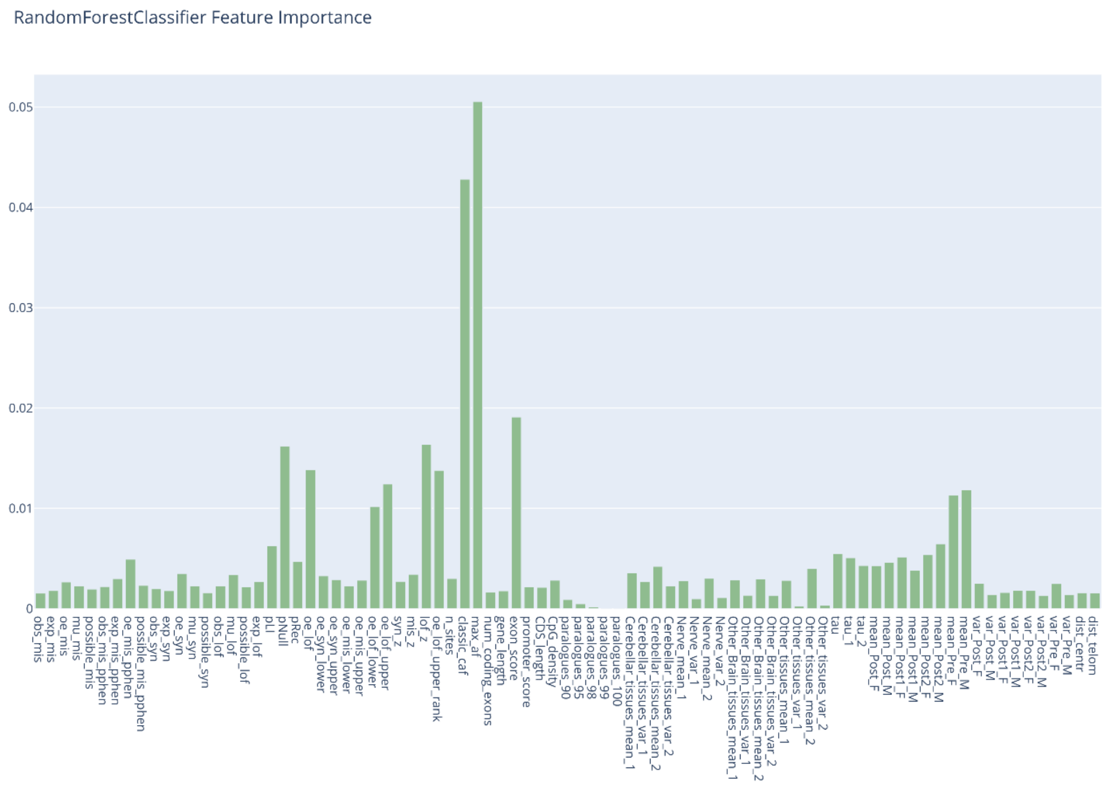

Supplementary Fig. 5: Feature importance for the the five machine learning classifiers with highest mcc: AdaBoostClassifier (**a**), BaggingClassifier (**b**), LinearSVC (**c**), MLPClassifier (**d**) and RandomForestClassifier (**e**). Source data are provided as a Source Data file.

a

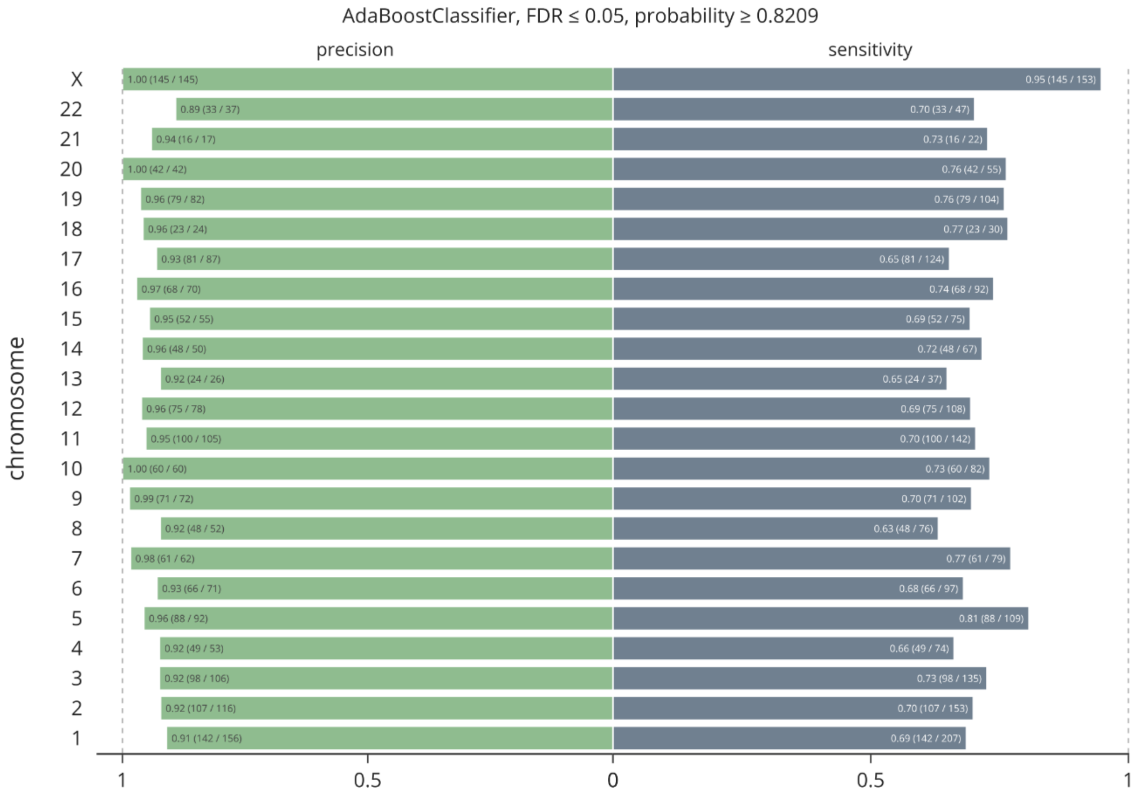

b

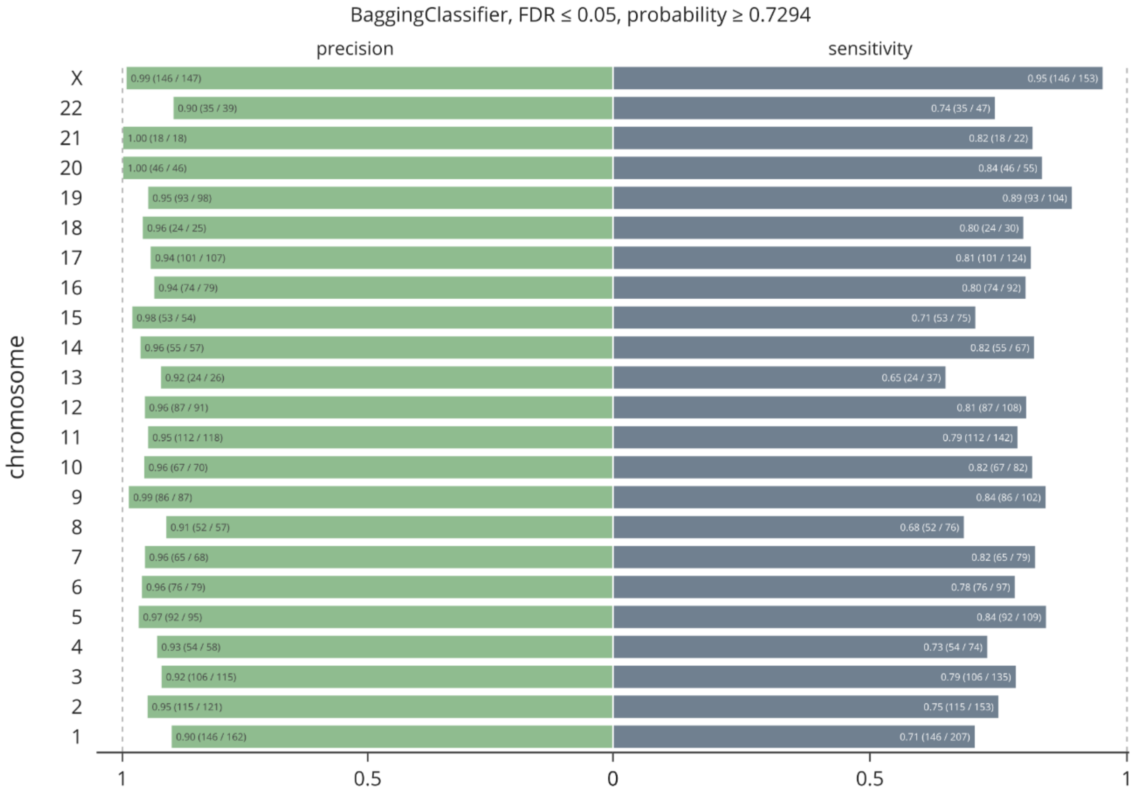

Supplementary Fig. 6 (continue next page)

c

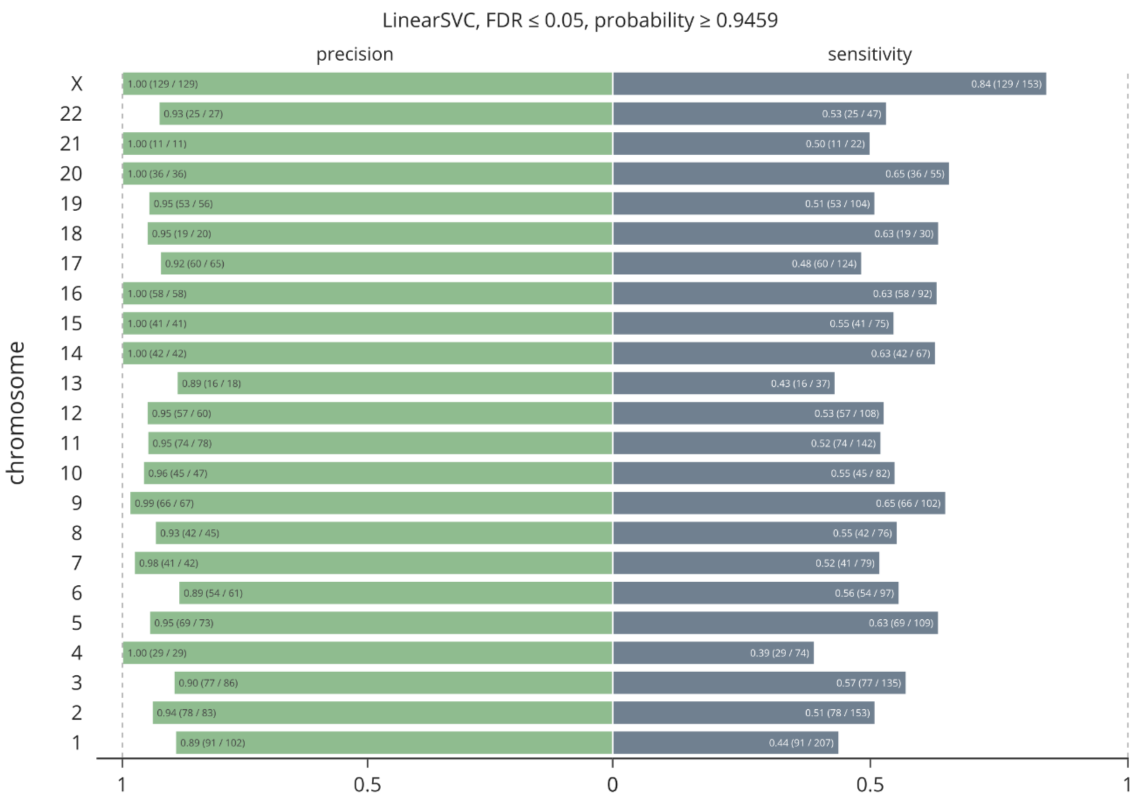

d

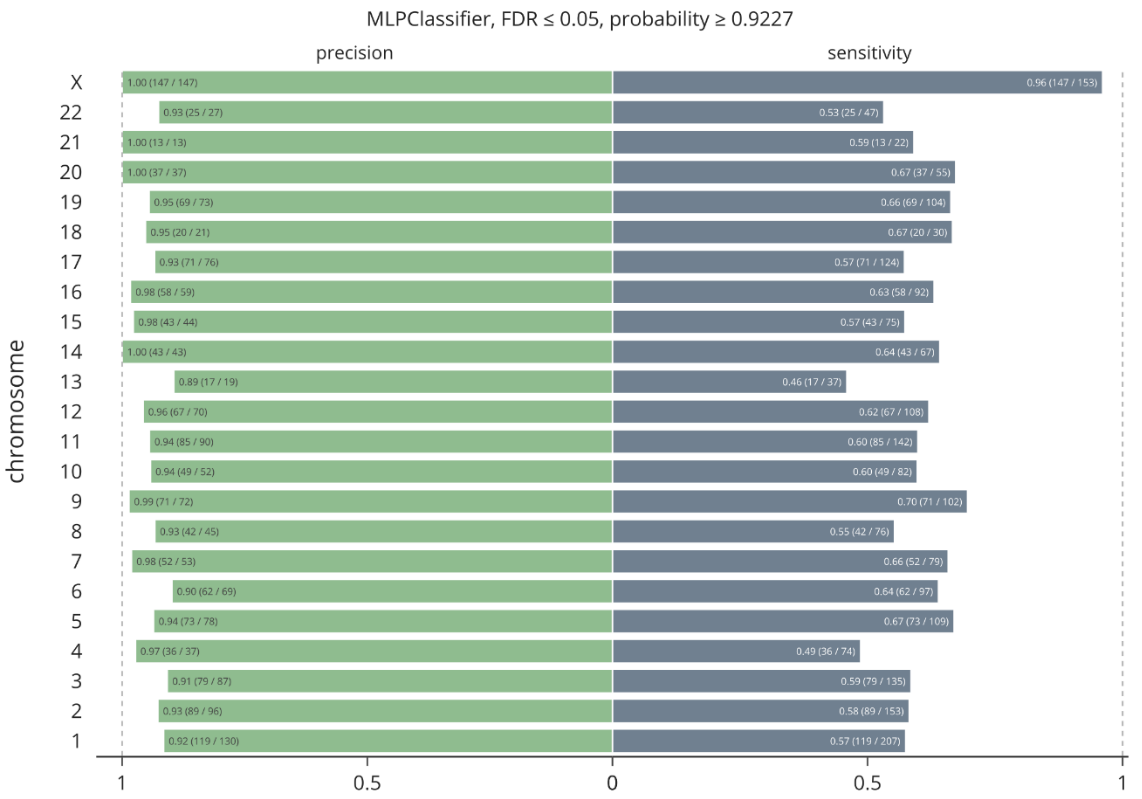

Supplementary Fig. 6 (continue next page)

e

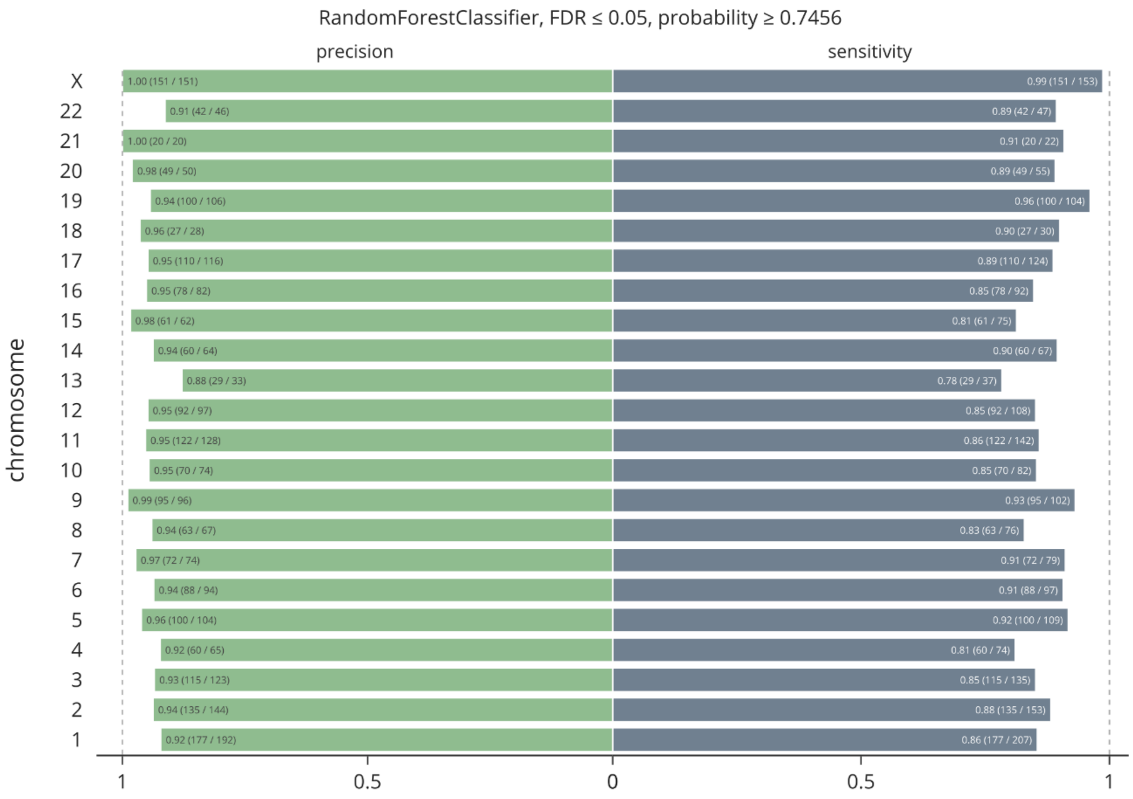

Supplementary Fig. 6: Sensitivity and precision metrics across chromosomes for the five machine learning classifiers with highest mcc: AdaBoostClassifier (**a**), BaggingClassifier (**b**), LinearSVC (**c**), MLPClassifier (**d**) and RandomForestClassifier (**e**). Source data are provided as a Source Data file.

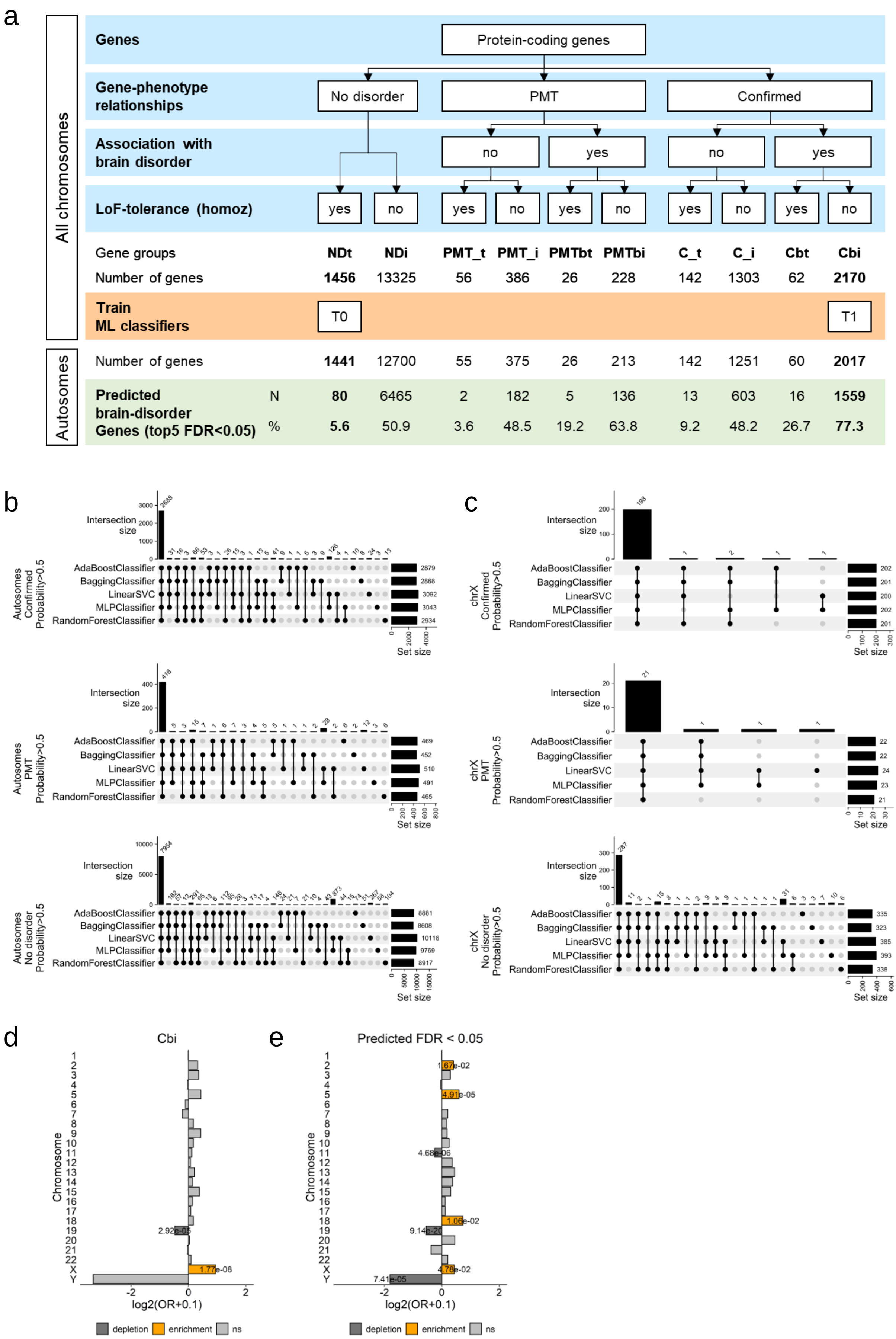

Supplementary Fig. 7 (legend next page)

Supplementary Fig. 7: Machine learning predictions on chrX compared to autosomes. a, Protein-coding genes were pre-classified into 10 subgroups based on 1) the type of associations with disorders and/or traits (confirmed, PMT, no-disorder), 2) the association with a brain disorder and 3) the tolerance to loss-of-function (LoF) homozygous variants. Two classes were used to train the machine learning classifiers: Cbi (confirmed brain-disorder associated genes that are LoF-intolerant; value 1.0) and NDt (no-disorder genes tolerant to LoF mutations; value 0). We show number and fraction of predicted genes (FDR<0.05) for each of the 10 classes for autosomes. Data underlying this scheme can be found in Supplementary Data 6. **b and c**, Upset plot showing the number of genes predicted by each of the top five ML classifiers (set size) and the number of genes shared between classifiers (intersection size) for confirmed, PMTs and no-disorder genes on autosomes (**b**) and on chrX (**c**). **d and e,** Per chromosome enrichment/depletion of Cbi genes (**d**) and correctly predicted Cbi genes at FDR<0.05 (**e**). Fisher’s test (two-sided) followed by Bonferroni correction for multiple testing. Source data are provided as a Source Data file.

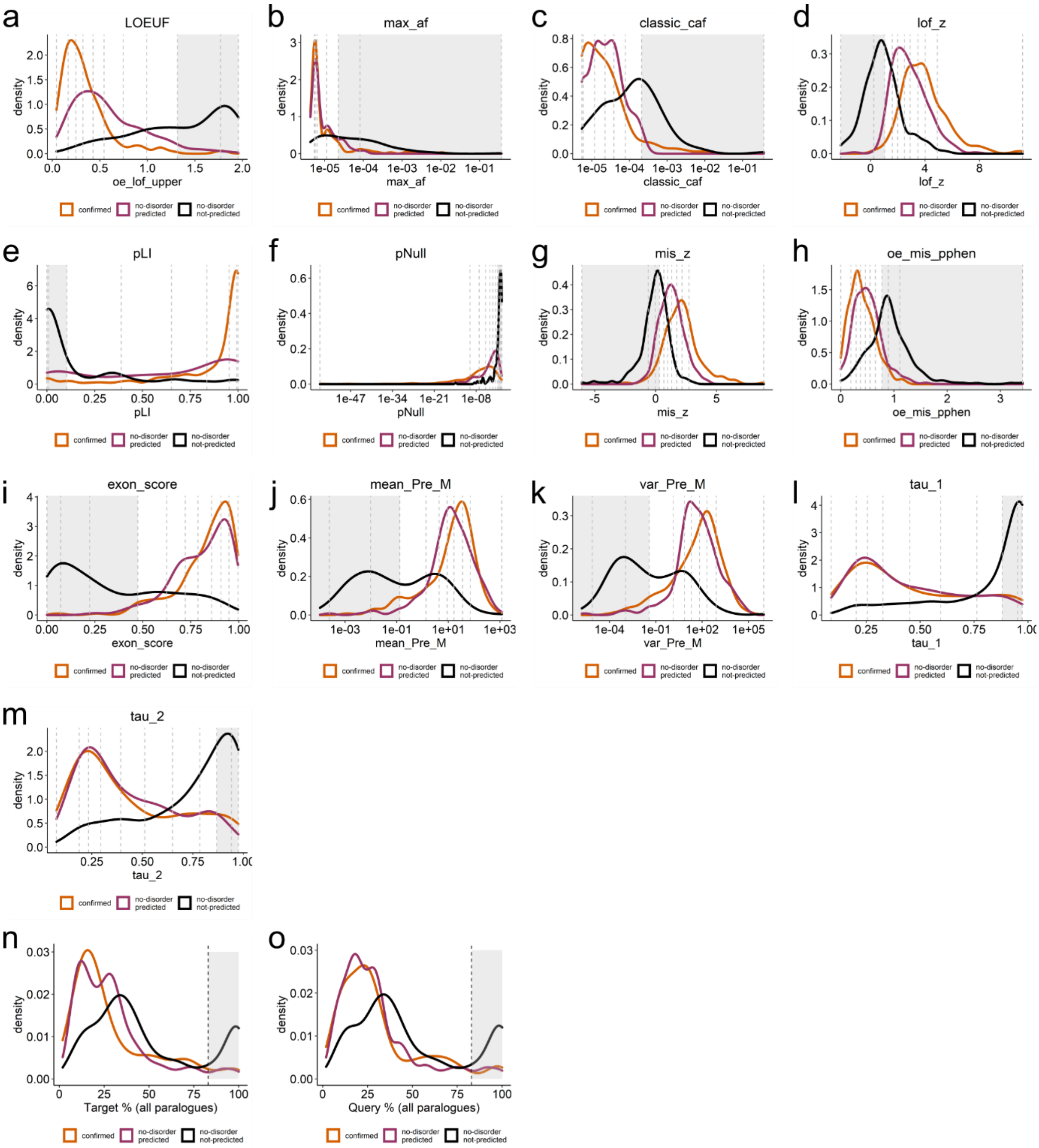

Supplementary Fig. 8: Distribution of relevant features used as input for the machine learning classifiers. a-m, Density plots showing the distribution LOEUF, misZ, exon_score and other important features according to gene group and/or ML predicted status for chrX genes. Vertical dashed lines separate deciles of the overall distribution. Grey areas depict deciles for which no-disorder not-predicted genes are enriched (Fisher's test (two-sided) followed by Bonferroni correction for multiple testing, across both features and deciles). **n and o,** Density plots showing the distribution of target (**n**) and query (**o**) percentage of identity for paralogues of chrX genes according to gene group and/or ML predicted status for chrX genes. Vertical dashed lines depict the 95th percentile of the overall distribution (81.4 and 83.9% for target and query percentages of identity, respectively), and grey areas mark paralogues above the 95th percentile. **a-o,** Genes associated with at least one monogenic disorder (confirmed genes, orange), no-disorder predicted by ML as being disease-associated (no-disorder predicted, purple), and no-disorder not-predicted genes (black). Source data are provided as a Source Data file.

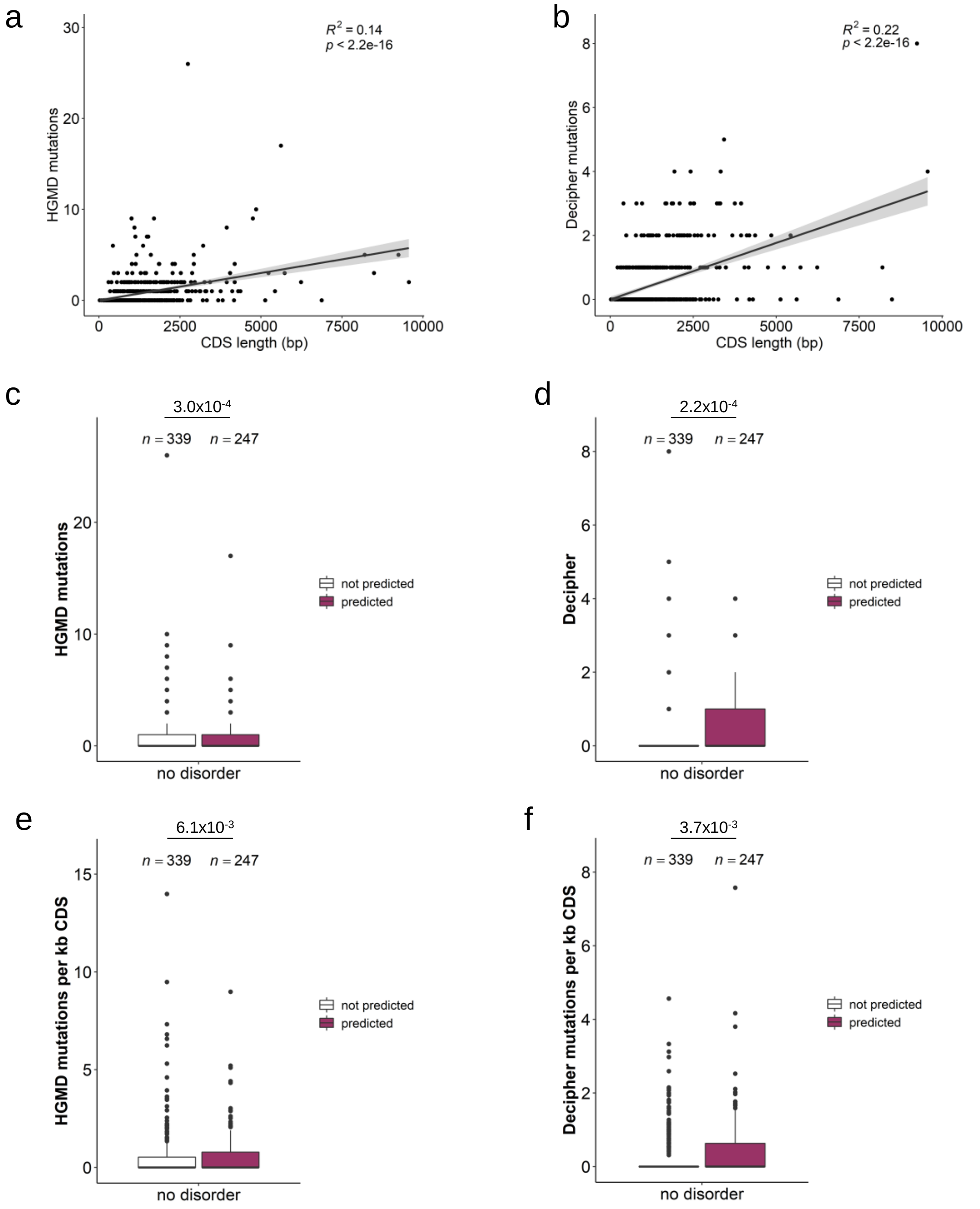

Supplementary Fig. 9: Known point mutations in no-disorder genes. a-b, Scatter plots showing the correlation between the coding-sequence (CDS) size and the number of known HGMD. Pearson's correlation (two-sided) method was used. Shaded area corresponds to the confidence interval of 0.95. (**a**) and DECIPHER (**b**) mutations. **c-d**, Boxplot showing the number of known mutations reported on HGMD (**c**) and DECIPHER (**d**) for no-disorder genes according to their predicted status. **e-f**, Boxplot showing the number of known mutations normalized by coding-sequence (CDS) length due to the small correlation between the two variables that were reported on HGMD (**e**) and DECIPHER (**f**) for no-disorder genes according to their predicted status. **c-f,** Box plot elements are defined as follows: center line: median; box limits: upper and lower quartiles; whiskers: 1.5× interquartile range; points: outliers. Mann-Whitney U test (two-sided) followed by Bonferroni correction for multiple testing. Source data are provided as a Source Data file.

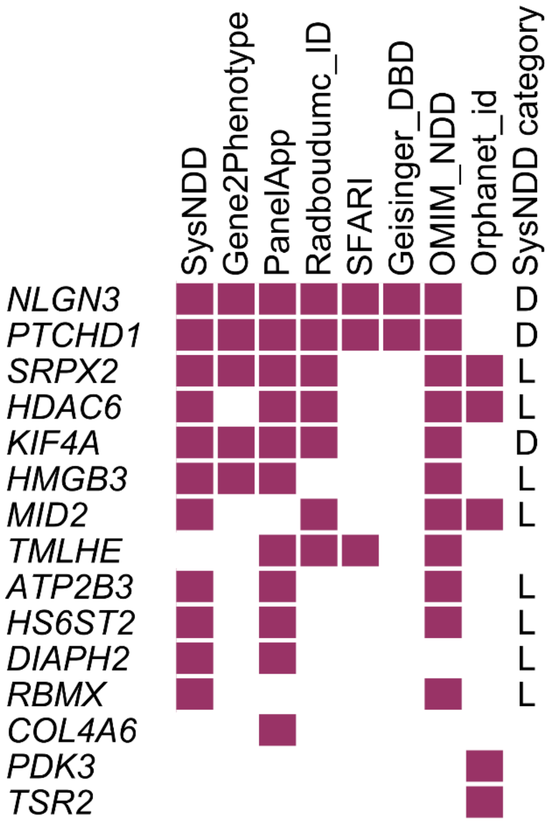

Supplementary Fig. 10: Predicted PMT genes present in expert curated NDD gene databases. Genes in SysNDD are classified as D (definite NDD gene) or L (limited evidence as NDD gene). Data underlying this scheme can be found in Supplementary Data 8.

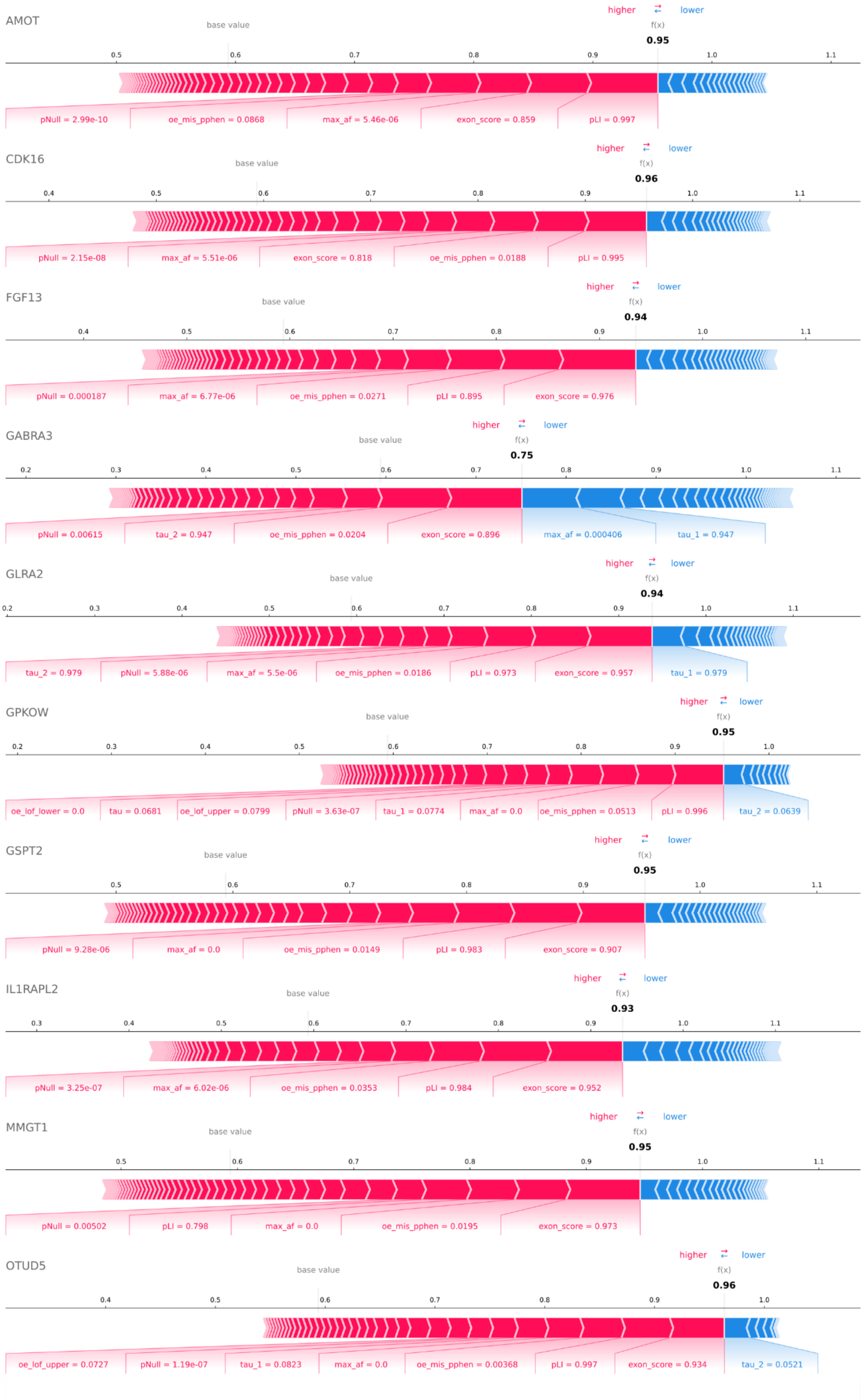

Supplementary Fig. 11 (continue next page)

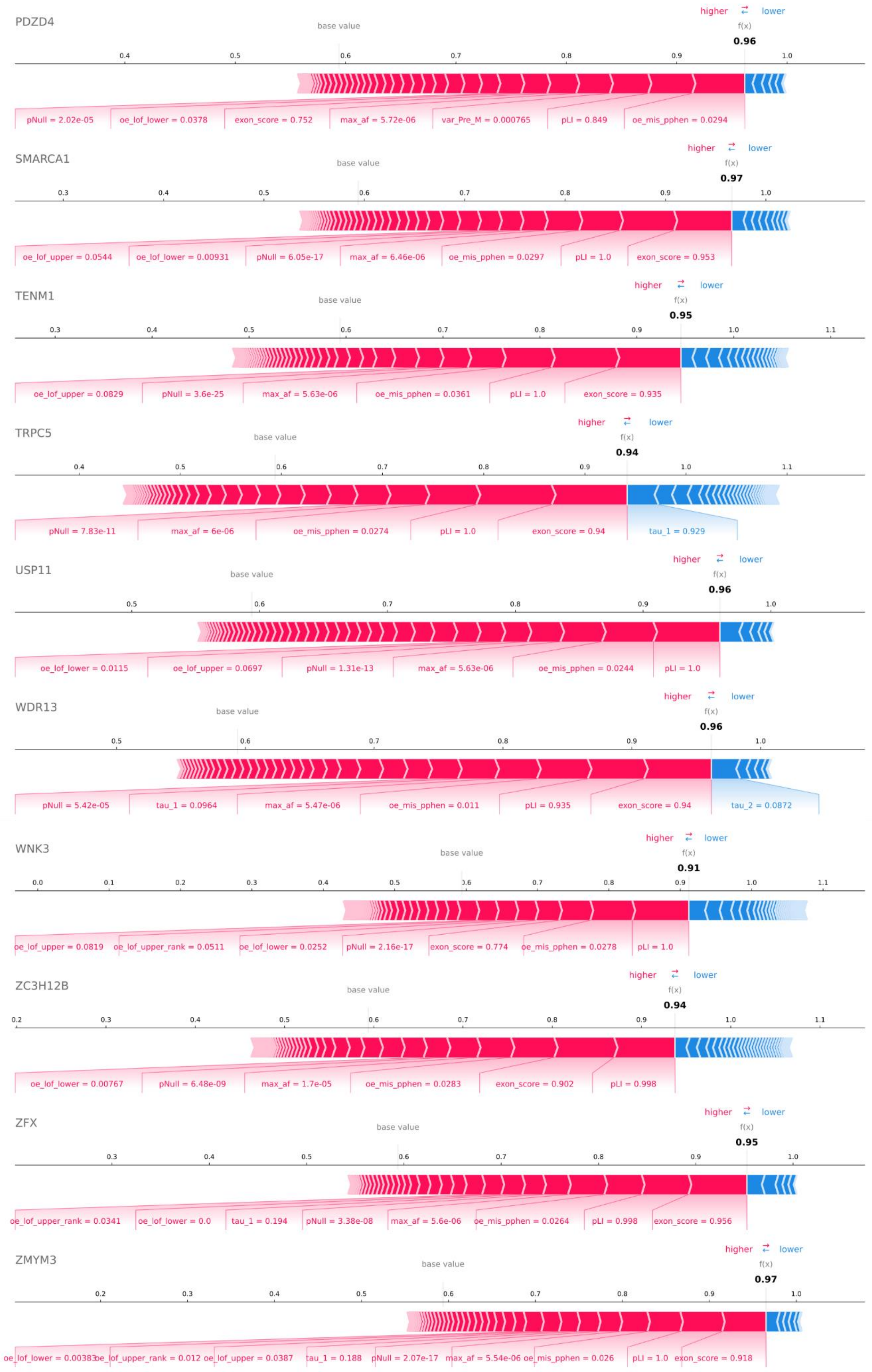

Supplementary Fig. 11: Force plot visualization of the prediction explanations for 20 selected genes. Features pushing the prediction higher are shown in red, and those pushing the prediction lower are in blue. Plots for all chrX genes are available in a Github repository.[1]

Supplementary References

1. Schröder C, Leitão E, Depienne C. Systematic analysis and prediction of genes associated with monogenic disorders on human chromosome X. christopher-schroeder/chrX_gene_predictions. https://doi.org/10.5281/zenodo.7031826 (2022).
