# Supplementary material for: Systematic analysis and prediction of genes associated with monogenic disorders on human chromosome X
Source: Nat Commun. 2022 Nov 2;13:6570. doi: 10.1038/s41467-022-34264-y (PMC9630267; doi:10.1038/s41467-022-34264-y)
Supplement: Supplementary file 3 — Description of Additional Supplementary Files [file 41467_2022_34264_MOESM3_ESM.pdf]

Description of Additional Supplementary Files

Supplementary Data 1-11. Excel file containing Supplementary Data 1-11 in individual sheets:

Supplementary Data 1. Gene set definitions

Supplementary Data 2. Annotations of protein-coding genes on chrX

Supplementary Data 3. Terms/sentences used as synonymous in the OMIM Advanced Search

Supplementary Data 4. Gene annotations for autosomes and sex chromosomes

Supplementary Data 5. Description of data columns on Supplementary Table 6 relative to the machine learning classifiers

Supplementary Data 6. Data related to the machine learning classifiers

Supplementary Data 7. Characteristics of the 25 machine learning models

Supplementary Data 8. Reported mutations in no-disorder and PMT genes and presence in NDD gene lists

Supplementary Data 9. Number of damaging variants (truncating or CADD≥25) in two published studies and two additional cohorts of patients with developmental disorders

Supplementary Data 10. Damaging variants (truncating or CADD≥25) in 13 selected genes

Supplementary Data 11. Damaging variants (truncating or CADD≥25) in CDK16 and TRPC5
