# Supplementary material for: Systematic analysis and prediction of genes associated with monogenic disorders on human chromosome X
Source: Nat Commun. 2022 Nov 2;13:6570. doi: 10.1038/s41467-022-34264-y (PMC9630267; doi:10.1038/s41467-022-34264-y)
Supplement: Supplementary file 5 — Reporting Summary [file 41467_2022_34264_MOESM5_ESM.pdf]

nature portfolio

Corresponding author(s): Christel Depienne

Last updated by author(s): Sep 27, 2022

Reporting Summary

Nature Portfolio wishes to improve the reproducibility of the work that we publish. This form provides structure for consistency and transparency in reporting. For further information on Nature Portfolio policies, see our Editorial Policies and the Editorial Policy Checklist.

Statistics

For all statistical analyses, confirm that the following items are present in the figure legend, table legend, main text, or Methods section.

n/a	Confirmed	
☐	☒	The exact sample size (*n*) for each experimental group/condition, given as a discrete number and unit of measurement
☒	☐	A statement on whether measurements were taken from distinct samples or whether the same sample was measured repeatedly
☐	☒	The statistical test(s) used AND whether they are one- or two-sided<br>*Only common tests should be described solely by name; describe more complex techniques in the Methods section.*
☐	☒	A description of all covariates tested
☐	☒	A description of any assumptions or corrections, such as tests of normality and adjustment for multiple comparisons
☐	☒	A full description of the statistical parameters including central tendency (e.g. means) or other basic estimates (e.g. regression coefficient) AND variation (e.g. standard deviation) or associated estimates of uncertainty (e.g. confidence intervals)
☐	☒	For null hypothesis testing, the test statistic (e.g. *F*, *t*, *r*) with confidence intervals, effect sizes, degrees of freedom and *P* value noted<br>*Give P values as exact values whenever suitable.*
☒	☐	For Bayesian analysis, information on the choice of priors and Markov chain Monte Carlo settings
☒	☐	For hierarchical and complex designs, identification of the appropriate level for tests and full reporting of outcomes
☐	☒	Estimates of effect sizes (e.g. Cohen's *d*, Pearson's *r*), indicating how they were calculated

Our web collection on statistics for biologists contains articles on many of the points above.

Software and code

Policy information about availability of computer code

Data collection	Patch-clamp data acquisition (TRPC5 channel currents) was performed with the Axon Clampex 11 Software (Axon™ pCLAMP™ Software Suite).
Data analysis	The following publicly available softwares / packages were used to perform analyses:<br>Python 3.10.5 and packages scikit-learn 1.0.2, pandas 1.4.2, numpy 1.22.3, repackage 0.7.3, plotly 5.8.0, python-kaleido 0.2.1, shap 0.41.0 and matplotlib-base 3.5.2. R 4.2.0 and packages readr 2.1.2, dplyr 1.0.9, readxl 1.4.0, stringr 1.4.0, tidyr 1.2.0, ggplot2 3.3.6, biomaRt 2.52.0, seqinr 4.2-16, matrixStats 0.62.0, tibble 3.1.7, purr 0.3.4, GenomicScores 2.8.2, phastCons100way.UCSC.hg38 3.7.1, rstatix 0.7.0, ggpubr 0.4.0, karyoploteR 1.22.0, UniprotR 2.2.1, ggforce 0.3.3, grid 4.2.0, ggfortify 0.4.14, factoextra 1.0.7, gplots 3.1.3, gridExtra 2.3, cluster 2.1.3, scales 1.2.0, eulerr 6.1.1, RColorBrewer 1.1-3, Caret 6.0-92, Rcompanion 2.4.15, ggpmisc 0.4.7, UpSetR 1.4.0, ComplexHeatmap 2.12.0 and regioneR 1.28.0.<br>For patch-clamp, data analysis was done using Axon Clampfit 11 Software (Axon™ pCLAMP™ Software Suite), OriginPro 2017 and SigmaPlot 12.5.

For manuscripts utilizing custom algorithms or software that are central to the research but not yet described in published literature, software must be made available to editors and reviewers. We strongly encourage code deposition in a community repository (e.g. GitHub). See the Nature Portfolio guidelines for submitting code & software for further information.

Data

Policy information about availability of data

All manuscripts must include a data availability statement. This statement should provide the following information, where applicable:

- Accession codes, unique identifiers, or web links for publicly available datasets
- A description of any restrictions on data availability
- For clinical datasets or third party data, please ensure that the statement adheres to our policy

Source data are provided with this paper. The data generated in this study are provided in the Supplementary Information, Supplementary Data or Source Data files. We used publicly available data from HUGO Gene Nomenclature Committee (HGNC) Database (https://www.genenames.org/), Online Mendelian Inheritance in Man (OMIM) (https://www.omim.org/), gnomAD v2.1.1 (https://gnomad.broadinstitute.org/), GTEx Portal (https://gtexportal.org/home/), the Developmental Transcriptome dataset from BrainSpan (https://www.brainspan.org/), Ensembl (https://www.ensembl.org/index.html), UCSC Table Browser (https://genome.ucsc.edu/cgi-bin/hgTables) Uniprot (https://www.uniprot.org/), DECIPHER (https://www.deciphergenomics.org/), SysNDD database (https://sysndd.dbmr.unibe.ch/), and CADD - Combined Annotation Dependent Depletion (https://cadd.gs.washington.edu/). In addition, two published datasets from Martin et al. 2021 and Kaplanis et al. 2020 and variants from the Human Gene Mutation Database (HGMD) professional database (commercially distributed by Qiagen) were used.

Field-specific reporting

Please select the one below that is the best fit for your research. If you are not sure, read the appropriate sections before making your selection.

☒ Life sciences ☐ Behavioural & social sciences ☐ Ecological, evolutionary & environmental sciences

For a reference copy of the document with all sections, see nature.com/documents/nr-reporting-summary-flat.pdf

Life sciences study design

All studies must disclose on these points even when the disclosure is negative.

Sample size	We included in the study all approved coding genes on chr X or on autosomes (source: HGNC). We retrieved all point variants (SNVs or indels) reported in specific genes from specific sources: HGMD, Decipher. Furthermore, we also included genetic data from patients from two different cohorts. The respective numbers corresponds to patients for whom data was available.
Data exclusions	Noncoding genes (i.e. coding genes encoding lncRNA or other RNA types not known to be translated into proteins) were not included. Furthermore, MED14OS was excluded from the analysis because it was indicated as "Product of a dubious CDS prediction".
Replication	We used different machine learning tools to predict genes associated with disorders and checked that the list of predicted genes is largely overlapping using the best five tools.<br>Regarding the experiments displayed in Figure 8e-g, two different data acquisitions performed at different times (2021 and 2022) on the same platform led to similar results.
Randomization	Participants were from affected cohorts. They were not allocated to any experimental groups.
Blinding	The investigator performing the patch-clamp experiments was blinded to the TRPC5 channel group (WT/ mutant) being recorded. The group was revealed for analysis.

Reporting for specific materials, systems and methods

We require information from authors about some types of materials, experimental systems and methods used in many studies. Here, indicate whether each material, system or method listed is relevant to your study. If you are not sure if a list item applies to your research, read the appropriate section before selecting a response.

Materials & experimental systems

n/a	Involved in the study	
☒	☐	Antibodies
☐	☒	Eukaryotic cell lines
☒	☐	Palaeontology and archaeology
☒	☐	Animals and other organisms
☐	☒	Human research participants
☒	☐	Clinical data
☒	☐	Dual use research of concern

Methods

n/a	Involved in the study	
☒	☐	ChIP-seq
☒	☐	Flow cytometry
☒	☐	MRI-based neuroimaging

Eukaryotic cell lines

Policy information about cell lines

Cell line source(s)	HEK-293 cells (Human cells from embryonic kidney), initially bought from ATCC
Authentication	We did not use any authentication process other than initially buying the cells from a commercial source
Mycoplasma contamination	HEK-293 cells were negative for mycoplasma when received. The cell culture facility performs regular testing of mycoplasma to detect contaminated cell lines. HEK-293 cells specifically used in patch-clamp recordings were not tested for mycoplasma contamination.
Commonly misidentified lines (See ICLAC register)	We haven't used any commonly misidentified cell lines.

Human research participants

Policy information about studies involving human research participants

Population characteristics	Two independent cohorts of patients with neurodevelopmental disorders (NDD) were included in the study.
Recruitment	The first cohort is a French cohort of patients with intellectual disability (ID) referred to the APHP reference center (Paris, France). The second cohort is an American cohort of patients with agenesis of the corpus callosum (AgCC) referred to UCSF (San Francisco, USA).
Ethics oversight	Data collection sites had study protocols approved by the local Institutional Review Boards (IRB) including INSERM (RBM C12-06), UCSF, and Ethik Kommission der Medizinischen Fakultät der Universität Duisburg-Essen.

Note that full information on the approval of the study protocol must also be provided in the manuscript.
